# NAM—help or burden? Intercultural evaluation of parental stress caused by nasoalveolar molding: a retrospective multi-center study

**DOI:** 10.1007/s00784-021-03850-7

**Published:** 2021-03-04

**Authors:** Maximilian Roth, Daniel Lonic, Florian D. Grill, Lucas M. Ritschl, Denys J. Loeffelbein, Klaus-Dietrich Wolff, Lien-Shin Niu, Betty Chien-Jung Pai, Lukas Prantl, Andreas Kehrer, Paul I. Heidekrüger, Andrea Rau, Lun-Jou Lo

**Affiliations:** 1 Department of Oro-Maxillofacial Surgery, University Hospital rechts der Isar, Technical University of Munich, Munich, Germany; 2 Department of Cranio- and Maxillo-facial Surgery, University Hospital Regensburg, Franz-Josef-Strauß-Allee 11, D-93053 Regensburg, Germany; 3 Centre of Plastic, Aesthetic, Hand and Reconstructive Surgery, University Hospital Regensburg, Regensburg, Germany; 4 Department of Plastic and Reconstructive Surgery and Craniofacial Research Center, Chang Gung Memorial Hospital, Chang Gung University, 5, Fu-Shin Street, Kwei Shan, Taoyuan, Taiwan 333; 5 Department of Plastic and Reconstructive Surgery, Helios Hospital München West, Teaching Hospital of Ludwigs-Maximilian-Universität München, Munich, Germany; 6 MFACE | KieferGesichtsZentrum München, Munich, Germany; 7 Department of Oral and Maxillofacial Surgery, Helios Hospital München West, Teaching Hospital of Ludwig-Maximilian-Universität München, Munich, Germany; 8 Craniofacial Research Center, Chang Gung Memorial Hospital, Chang Gung University, Taoyuan, Taiwan; 9 Department of Craniofacial Orthodontics and Craniofacial Research Center, Chang Gung Memorial Hospital, Taoyuan, Taiwan; 10 Department of Oral and Cranio-Maxillofacial Surgery, University Hospital Erlangen, Erlangen, Germany

**Keywords:** Nasoalveolar molding, Intercultural evaluation, Parental stress, Burden, Cleft lip palate, Questionnaire

## Abstract

**Objectives:**

Nasoalveolar molding (NAM) was developed to facilitate easier treatment and better outcomes for cleft lip and palate (CLP) patients. The aim of this study was to investigate the parental burden and possible intercultural differences of this treatment modality, which is often argued to burden parents to an extraordinary amount.

**Materials and methods:**

Standardized questionnaires (available in English, Mandarin, and German) with 15 non-specific and 14 NAM-specific items to be retrospectively answered by Likert scales by parents of unilateral CLP patients with completed NAM treatment.

**Results:**

The parents of 117 patients from two treatment centers in Taiwan and Germany were included. A very high level of overall satisfaction was found in both countries with significant intercultural differences in prenatal parent information, feeding problems, dealing with 3rd party’s perception, and experienced personal effort.

**Conclusion:**

NAM is an effective treatment tool for children’s CLP deformities and their caregivers in overcoming the feeling of helplessness. Intercultural differences may be due to infrastructural reasons, cultural attitudes and habits, or different public medical education.

**Clinical relevance:**

In addition to facilitating easier surgical treatment, NAM can be seen as a powerful coping strategy for parents dealing with a CLP deformity of their child and does not seem to burden them extraordinarily.

## Introduction

**Fig. 1:**
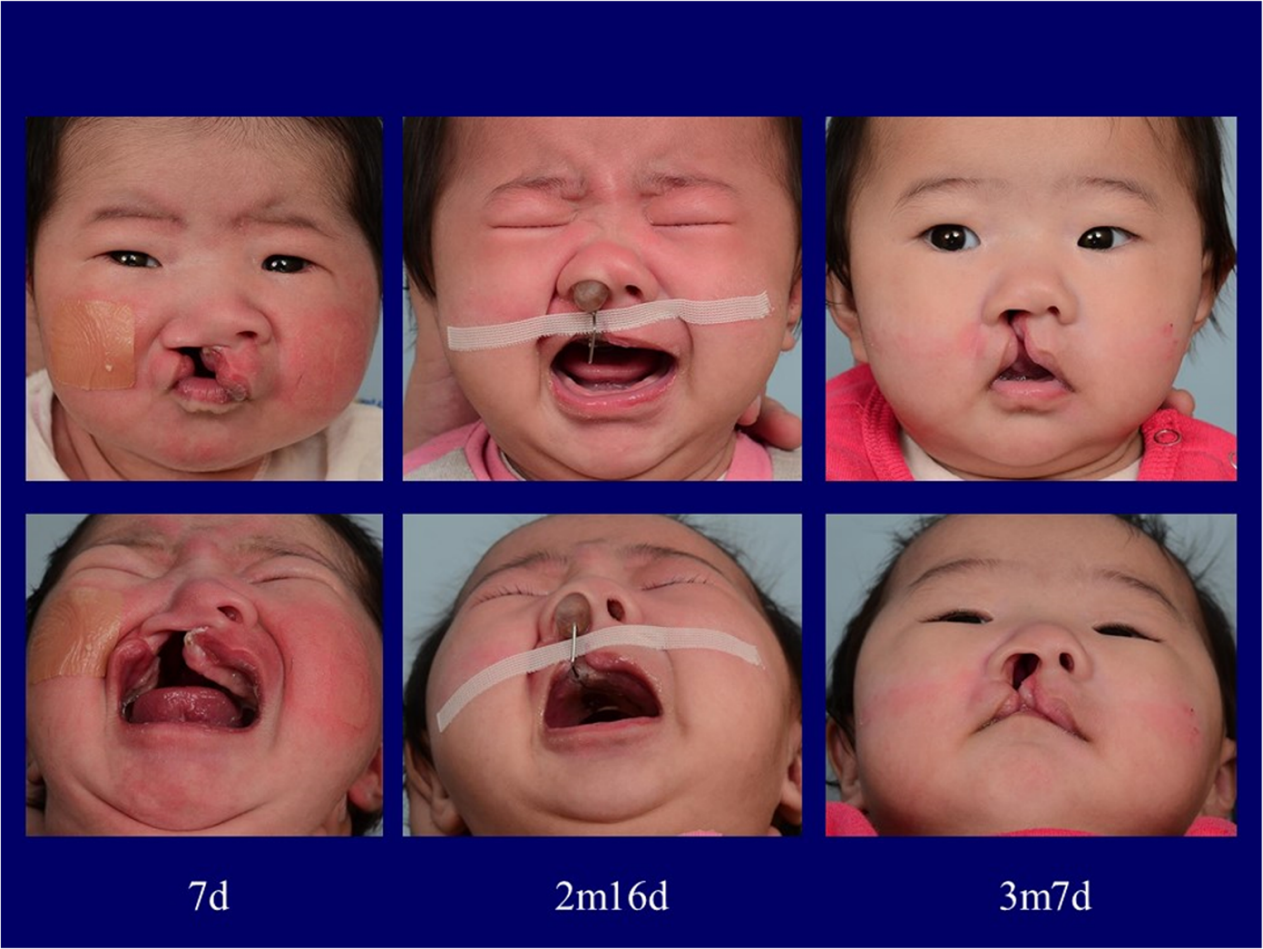
CLP patient prior to treatment (age 7 days), during treatment with NAM appliance and lip taping (age 2 months and 16 days), and post treatment (age 3 months and 7 days)

Cleft lip and palate (CLP) is one of the most frequent congenital deformities worldwide [1]. Among the several surgical issues of this entity, the functional and aesthetic reconstruction of the deformed cleft nose still poses one of the biggest challenges. Since its introduction over 20 years ago, nasoalveolar molding (NAM) has been constantly refined to facilitate easier treatment and better outcomes for cleft lip and nose deformity patients. First introduced by Grayson in the early 1990s [2, 3], NAM takes advantage of the high plasticity of neonatal cartilage in the early postnatal period [4] by using functional acrylic plates in combination with extraoral taping and nasal taps to guide maxillary and nasal shape and growth (Fig. 1). Correct and meticulous timing of the treatment steps is essential in cleft therapy [5–7]. Therefore, NAM treatment needs to be started early in the first days of life and continued with weekly management until the surgical lip repair at the age of approximately 3 months. NAM was shown to effectively reduce alveolar cleft width, improve nasal symmetry and, in bilateral clefts, realign the premaxilla, and lengthen the columella [7–13]. Since treatment compliance and motivation is of utmost importance and cannot be provided by the small patients themselves, their caregivers are responsible for the daily handling of the NAM plate. Due to obvious infrastructural issues [14] such as frequent and time-consuming appointments or associated higher treatment costs, several promising attempts have been reported to simplify the techniques, lower the costs, and shorten appointments or lower their frequency [15–17]. However, many different aspects can contribute to psychological burden during the time of the treatment, some of which are based on cultural- and gender-related perspectives.

The aim of this study was to investigate possible intercultural and gender differences in parents’ perception of the psychological burden of NAM treatment. The cooperation combined data from two treatment centers in Munich, Germany, and Taoyuan, Taiwan.

## Materials and methods

### Patients

Cleft databases from the Chang Gung Memorial Hospital, Taoyuan, Taiwan, and the University Hospital rechts der Isar, Munich, Germany, have been searched for patients with complete unilateral CLP who had been treated with NAM. The term “complete” referred to a cleft manifestation of the lip, alveolus, and palate. Approval was obtained by both local ethics committees (University Hospital rechts der Isar, number 92/15; Chang Gung Memorial Hospital, IRB 104-2216B) before questionnaires were sent to the patients’ parents. Mothers and fathers were asked to answer these questionnaires separately. Participation was completely voluntary, no participant was urged in any kind to reply, and all participants answering the questionnaires were of age 18 or above. Informed consent for study participation and for publication of images was obtained from the parents including online open-access publication. All methods were performed in accordance with the relevant guidelines and regulations.

### Questionnaires

The questionnaires were designed in interdisciplinary work with members of both centers involving maxillofacial surgeons, plastic surgeons, speech therapists, psychologists, and statisticians. The questionnaire consisted of two parts: the first general part contained 15 NAM-independent standardized items and one field for comments, and the second modality-specific part contained 14 NAM-specific standardized items and one more field for comments. These 29 items could each be answered by a Likert scale of five answer categories (1 = strong disagreement to 5 = strong agreement) [18]. Subsequently the questionnaire was translated into German and Mandarin and again validated by members of both teams.

For more information, see Table 1*.*

**Table 1:** Single-choice questionnaire cleft lip and palate

Question no.	Question
1	When I found out that my child would have cleft lip palate, I was concerned about the future
2	I was afraid that feeding my child would be difficult
3	I was afraid of other people’s reactions
4	The information about cleft lip palate given to me by my doctors before the birth were helpful and reassuring
5	After the birth of my child, I quickly learned to adapt to the special needs of my child with respect to feeding and care
6	In the first few days after the birth, I had to become accustomed to the appearance of my child
7	My child could be breastfed
8	Bottle-feeding was possible without problems
9	Feeding my child in his/her first year was generally problematical
10	The frequent visits to the doctor during my child’s first year were a burden
11	My child’s first year was difficult for me, and I often felt overwhelmed
12	In looking after my child, I was well supported by members of my social environment (family, friends, etc.)
13	Strangers often asked me about my child’s cleft lip and palate
14	I was able to deal well with strangers’ reactions concerning my child’s cleft lip palate
15	I benefited from conversations with other parents in the same situation
16	*field for comments*
	NAM-specific part:
17	Before beginning the treatment, I had doubts that I would be able to learn how to insert and attach the plate
18	The frequent fixing of the tapes and the insertion of the plate took me a lot of time
19	The insertion and attachment of the plate was easier than I had anticipated
20	During the 3-month treatment, I could see an improvement in the overall shape of the nose
21	During the 3-month treatment, I could see an improvement in the overall shape of the lip
22	I had the impression that the plate and the fixing tapes disturbed my child’s sleep and movement
23	During the treatment, my child often suffered from facial skin irritation (rashes)
24	My child was unable to drink without the plate
25	I had to remove the plate for my child to be able to drink
26	My partner and I took it in turns to insert the plate
27	I found it time-consuming to attend the weekly check-up appointments
28	I felt that I was well looked after during the 3-month treatment
29	I had the feeling that I was helping my child with the nasoalveolar molding therapy
30	All in all, I found the nasoalveolar molding therapy convincing
31	*Field for comments*

Answering options (Likert scale): strong disagreement (1), disagreement (2), neutral (3), agreement (4), strong agreement (5)

### Validation

Objectivity was achieved by observer independent questioning advising parents to self-respond to the mentioned questionnaires. Likert scales grant objectivity of analysis, while evaluating the grade of agreement or disagreement to simple statements ensures objectivity of interpretation. Language validation was performed by questionnaire design in English with subsequent translation and validation by a team of native speakers of German and Mandarin. Content validity was assured through our mentioned interdisciplinary team. Due to ethical concerns regarding intrusiveness and further parental burdening, consistent answers from the same family were assumed, and answers from mothers and fathers were tested for one-way randomized intra-class correlation (ICC), which further was interpreted according to Cicchetti [19] revealing good overall interrater reliability (except for questions #5 and #19).

### Data analysis

Data analysis was performed by using SPSS® for Mac 22.0.0 (IBM Corp.; Armonk, NY, USA). Descriptive statistics are given as mean values and standard errors. The *t*-test for independent samples was used for group comparisons between both centers. The level of significance was set at α ≤ 0.05.

Questions to be answered significantly different in international comparison were then validated: answers differing more than one grade regarding the Likert scale were assumed to be differing strongly, others negligibly [18].

## Results

### Database

- Klinikum rechts der Isar, Munich, Germany. The search of the database resulted in 18 patients with complete unilateral clefts. Twenty-eight of 36 sent questionnaires returned according to a return rate of 77.8%.
- Chang Gung Memorial Hospital, Taoyuan, Taiwan. Ninety-nine patients meeting the mentioned criteria were identified. Seventy-three of 198 sent questionnaires returned resulting in a return rate of 39.4%.

Gender distribution, mean parents’ age, and time between surgery and questioning are presented in Table 2. Mean parents’ age at time of questioning is in concordance with demographic data revealing no differences regarding parental age at the birth of firstborns.

**Table 2:** Gender distribution, mean parent’s age, and postprocedural observation

Origin of data	Parent						Parent’s age		Time between surgery and questioning (days)	
	Mother		Father		Total		Mean	SD	Mean	SD
	*n*	%	*n*	%	*n*	%				
Chang Gung Memorial Hospital	38	52.8%	34	47.2%	72	100.0%	35.6	6.6	602	687
Klinikum rechts der Isar	15	53.6%	13	46.4%	28	100.0%	35.7	7.0	737	551

### Intercultural differences

The results of the subgrouped analysis of the single questions are given in Table 3. Significant differences were found for the following groups of questions:

**Table 3:** Intercultural differences

Question	Origin of data	Mean	SE	*p*
#1	Chang Gung Memorial Hospital	3.65	.136	.146
	Klinikum rechts der Isar	4.04	.221	
#2	Chang Gung Memorial Hospital	3.82	.112	.894
	Klinikum rechts der Isar	3.79	.226	
#3	Chang Gung Memorial Hospital	3.33	.148	.218
	Klinikum rechts der Isar	3.71	.267	
#4	Chang Gung Memorial Hospital	4.28^1^	.080	.002*
	Klinikum rechts der Isar	3.17^1^	.312	
#5	Chang Gung Memorial Hospital	4.12^2^	.089	.018*
	Klinikum rechts der Isar	4.50^2^	.121	
#6	Chang Gung Memorial Hospital	3.59	.148	.108
	Klinikum rechts der Isar	3.04	.306	
#7	Chang Gung Memorial Hospital	2.59^1^	.142	.000*
	Klinikum rechts der Isar	1.14^1^	.143	
#8	Chang Gung Memorial Hospital	3.17^2^	.127	.004*
	Klinikum rechts der Isar	3.93^2^	.252	
#9	Chang Gung Memorial Hospital	3.17^1^	.130	.001*
	Klinikum rechts der Isar	2.14^1^	.245	
#10	Chang Gung Memorial Hospital	2.89	.138	.406
	Klinikum rechts der Isar	2.68	.193	
#11	Chang Gung Memorial Hospital	2.78	.125	.290
	Klinikum rechts der Isar	2.50	.227	
#12	Chang Gung Memorial Hospital	4.01	.085	.725
	Klinikum rechts der Isar	4.11	.248	
#13	Chang Gung Memorial Hospital	3.50^1^	.103	.001*
	Klinikum rechts der Isar	2.54^1^	.244	
#14	Chang Gung Memorial Hospital	3.79^2^	.097	.001*
	Klinikum rechts der Isar	4.46^2^	.158	
#15	Chang Gung Memorial Hospital	4.21^1^	.095	.004*
	Klinikum rechts der Isar	3.19^1^	.314	
#17	Chang Gung Memorial Hospital	3.44	.117	.115
	Klinikum rechts der Isar	2.93	.295	
#18	Chang Gung Memorial Hospital	3.29^2^	.129	.017*
	Klinikum rechts der Isar	2.68^2^	.212	
#19	Chang Gung Memorial Hospital	3.41	.113	.200
	Klinikum rechts der Isar	3.75	.234	
#20	Chang Gung Memorial Hospital	3.96	.102	.470
	Klinikum rechts der Isar	4.14	.234	
#21	Chang Gung Memorial Hospital	4.19	.091	.536
	Klinikum rechts der Isar	4.04	.227	
#22	Chang Gung Memorial Hospital	2.89^2^	.126	.004*
	Klinikum rechts der Isar	2.14^2^	.210	
#23	Chang Gung Memorial Hospital	3.09	.127	.564
	Klinikum rechts der Isar	3.25	.239	
#24	Chang Gung Memorial Hospital	3.51	.120	.772
	Klinikum rechts der Isar	3.61	.318	
#25	Chang Gung Memorial Hospital	2.32^2^	.128	.000*
	Klinikum rechts der Isar	1.36^2^	.201	
#26	Chang Gung Memorial Hospital	3.28	.143	.217
	Klinikum rechts der Isar	2.82	.334	
#27	Chang Gung Memorial Hospital	2.78	.122	.977
	Klinikum rechts der Isar	2.79	.301	
#28	Chang Gung Memorial Hospital	3.78^1^	.098	.000*
	Klinikum rechts der Isar	4.89^1^	.060	
#29	Chang Gung Memorial Hospital	4.43^2^	.077	.003*
	Klinikum rechts der Isar	4.82^2^	.074	
#30	Chang Gung Memorial Hospital	4.39	.082	.101
	Klinikum rechts der Isar	4.64	.128	

*p ≤ 0.05

^1^Strong difference

^2^Negligible difference

#### Prenatal information (Questions 4 and 15, Fig. 2)

Taiwanese (TWN) parents significantly felt to be more informed than the German (GER) ones regarding prenatal information given by doctors (#4: TWN 4.28 ±0.08 vs. GER 3.17 ±0.31; *p* < 0.01) and seem to profit more from information given by self-aid groups (#15: TWN 4.21 ±0.10 vs. GER 3.19 ±0.31; *p* < 0.01).

**Fig. 2:**
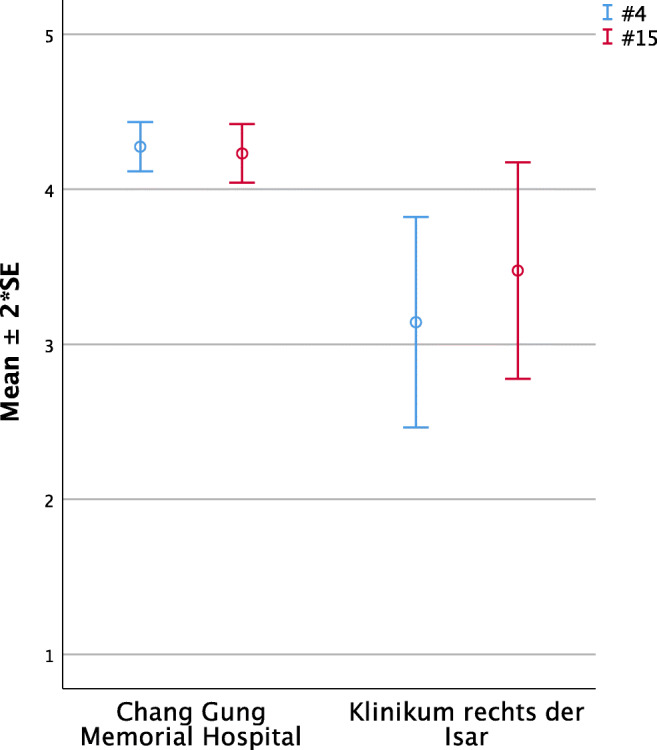
Significant intercultural differences in parental perception regarding prenatal information

#### Feeding (Questions 5, 7, to 9, Fig. 3)

German parents significantly felt to adapt quicker to the needs of their child with respect to feeding and care (#5 TWN 4.12 ±0.09 vs. GER 4.50 ±0.12; *p* = 0.02). Compared to their Taiwanese counterparts, German parents perceived more trouble in breast feeding (#7 TWN 2.59 ±0.14 vs. GER 1.14 ±0.14; *p* < 0.01) but less in bottle-feeding (#8 TWN 3.17 ±0.13 vs. GER 3.93 ±0.25; *p* = 0.02) and perceived less problems feeding their child in general (#9 TWN 3.17 ±0.13 vs. GER 2.14 ±0.25; *p* < 0.01).

**Fig. 3:**
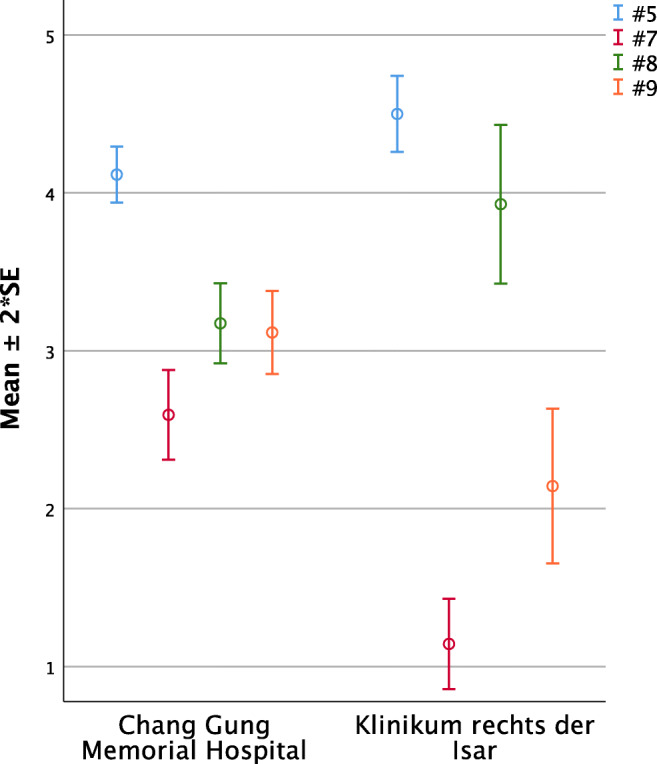
Significant intercultural differences in parental perception regarding feeding

#### 3rd Party’s perception (Questions 13 and 14, Fig. 4)

Taiwanese parents significantly felt to be asked more frequently about their child’s disease (#13 TWN 3.50 ±0.10 vs. GER 2.54 ±0.24; *p* < 0.01). While German parents answered to deal with others’ reactions very well, Taiwanese felt to deal significantly worse but still well (#14 TWN 3.79 ±0.10 vs. GER 4.46 ±0.16; *p* < 0.01).

**Fig. 4:**
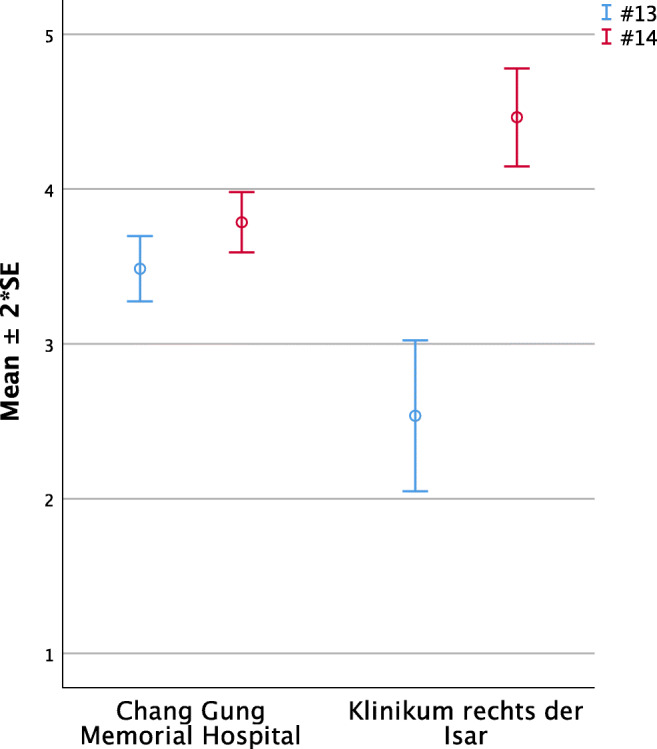
Significant intercultural differences in parental perception regarding 3rd party’s perception

#### Personal effort (Questions 18, 22, and 25, Fig. 5)

Parents stated to have spent moderate time for frequent tape-fixing and insertion of the plate with slight but significant differences, indicating that Taiwanese parents felt they spend more time for these activities than German parents (#18 TWN 3.29 ±0.13 vs. GER 2.68 ±0.21; *p* = 0.02). Similarly, Taiwanese parents perceived their child to be slightly more disturbed by the NAM device than German parents did, although both groups tended to see the device as rather non-disturbing (#22 TWN 2.89 ±0.13 vs. GER 2.14 ±0.21; *p* < 0.01). For feeding, German parents highly agreed to not being forced to remove the device, while Taiwanese parents had to remove it significantly more often (#25 TWN 2.32 ±0.13 vs. GER 1.36 ±0.20; *p* < 0.01).

**Fig. 5:**
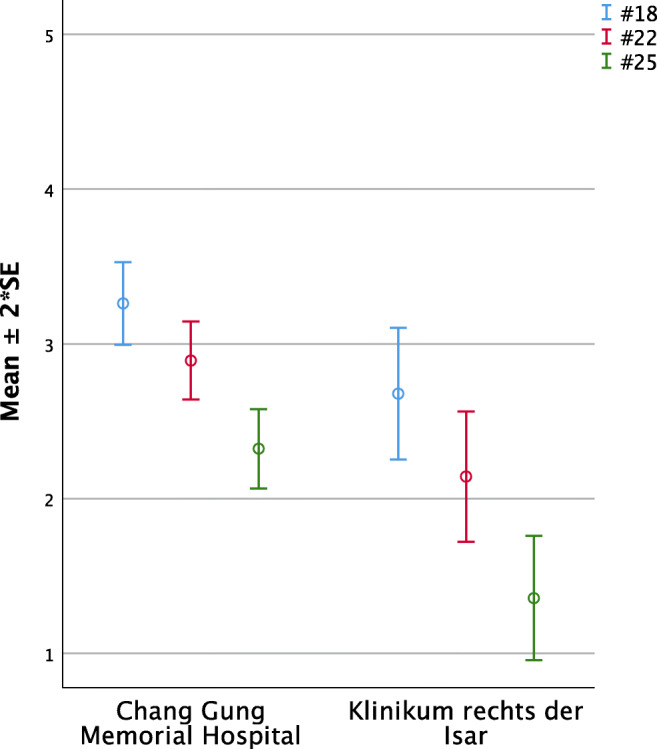
Significant intercultural differences in parental perception regarding personal effort

#### Overall satisfaction (Questions 28 to 30, Fig. 6)

Both groups felt to be well looked after, with the German parents even better (#28 TWN 3.78 ±0.10 vs. GER 4.89 ±0.06; *p* < 0.01). Regarding to having felt to help their child with NAM, both groups highly agreed with only a little lead of the German parents (#29 TWN 4.43 ±0.08 vs. GER 4.82 ±0.07; *p* < 0.01). Similarly, but not significantly different, Taiwanese and German parents answered the question whether they were convinced by NAM therapy very close to full agreement (#30 TWN 4.39 ±0.08 vs. GER 4.64 ±0.13; *p* = 0.10).

**Fig. 6:**
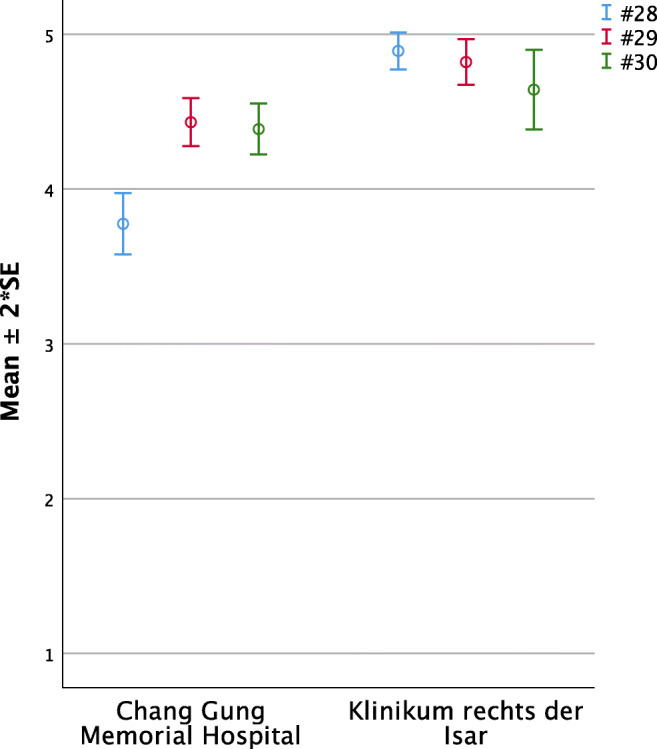
Significant intercultural differences in parental perception regarding overall satisfaction

#### Gender-related differences

Further analysis of possible gender-related differences in perception did not reveal any statistically significant differences neither for the whole collective nor in intercultural comparison.

#### Comment fields (items 16 and 31)

As there were only sporadic comments in the comment fields, the evaluation of this part of the questionnaire was omitted.

## Discussion

Despite great surgical progress and refined surgical techniques, the treatment of CLP deformities in newborn patients remains a difficult task. There have been many different proposals for techniques to presurgically narrow the cleft width and mold the soft tissue structures [8–12] with different degrees of invasiveness [2, 3, 8]. NAM can be seen as a non-invasive, semi-active, and very safe technique [2, 3, 20] and has proved to be very effective in narrowing cleft width and molding cartilaginous nasal structures with good long-term results [4, 9, 21, 22]. Nevertheless, critics often argue that NAM is a very burdening technique, especially by putting a lot of stress and responsibility on patients’ parents [23, 24], which may result in insufficient compliance [25]. Though the psychological effects of cleft deformities on parents are well described in the literature [26–28], the effect of presurgical treatment and its meaning as a coping strategy for parents has been investigated only rarely so far [24, 29]. Since it was already shown that approach-oriented coping strategies can be more useful for parents than avoidance-oriented ones [28], we believe NAM to be an effective treatment tool not only for the patients themselves but their parents as well. Nevertheless, this study did not investigate differences in the effectiveness of different techniques; thus, NAM might only be one tool among others to help parents cope with the situation.

The presented intercultural evaluation of two centers in Europe and Asia reveals more significant differences than one would expect while using the same treatment modality.

In this study, prenatal information about CLP showed to be significantly more helpful for parents from Taiwan than from Germany, which seems to reflect the use of self-aid groups helping parents to deal with the difficult situation. We suspect this to be due to infrastructural reasons [21, 30], as prenatal assessment of CLP deformities and care for CLP patients is centralized and closely connected in Taiwan, while in Germany, numerous comparable and smaller centers provide assessment and cleft care in a decentralized fashion. This may result in inconsistent strategies of prenatal assessment, assignment to CLP treatment centers, or even handling of CLP deformities before assigning affected parents to well organized self-aid groups.

### Questions 5, 7, to 9

Parents in Germany seem to slightly better adapt to their children’s needs in this situation, even though the difference is negligibly small and both groups felt to adapt to the special needs of their child very well overall. Several national and international studies among parents from both countries found breastfeeding to be more popular with German parents [20, 22, 31] than with Taiwanese parents [32–34]. In this study, we found German parents to perceive significantly more trouble with breastfeeding than Taiwanese. This may be due to the mentioned difference in breastfeeding frequency. Our results also indicate breastfeeding to be experienced as rather problematic in general for CLP patients’ parents. In contrast, bottle-feeding seemed rather unproblematic in both groups with negligibly small difference. This can be ascribed to the fact that the impairment of sucking and swallowing functions can be overcome by special bottle-feeding techniques and technical features [35, 36], e.g., by the use of a Haberman feeder. The combination of these three points may result in the perception of German parents that the general feeding difficulties are significantly less severe than experienced by Taiwanese parents.

### Questions 13 and 14

Taiwanese parents seem to be asked more frequently about their children’s deformity and thereby seem to feel under more peer pressure than German parents, which may result in a significantly better feeling of German parents when dealing with others’ reactions. To our knowledge, no study has been published so far directly comparing peer pressure caused by CLP deformities between German and Taiwanese patients or their parents. Yet similar findings for other cultural groups pointed out intercultural differences regarding general attitudes towards congenital deformities [26, 37–40]. This may explain the findings in this study and could emphasize the importance of parental education in order to develop better coping strategies on one hand [26] or on the other even the importance of further community health education[38, 39].

### Questions 18, 22, and 25

Even though there seems to be some significant differences in the perception of handling issues of the NAM devices in both groups, these intercultural differences are rather small. In general, handling does not seem to be problematic but rather easy and uncomplicated.

### Questions 28 to 30

Even though NAM remains a time- and cost-intensive and therefore to some extent burdening technique [14], generally parents from both cleft centers were highly satisfied and convinced by NAM, except for one minor difference regarding their support during NAM therapy. German parents felt significantly better supported, which could possibly represent one of the disadvantages of centralized and specialized high-volume health care (Taiwan) in contrast to very personal and small but decentralized teams (Germany). To support this thesis, this study would need to be expanded to more than only these two treatment centers.

The results of this study can help to emphasize certain support strategies to lessen the psychological burden of CLP patient parents during NAM therapy. For instance, the model of Taiwanese support groups could be implemented into scientifically curated digital platforms to overcome the shortcomings of decentralized care in other parts of the world. On the other hand, issues like the perception of cleft patients in the general public could also be positively influenced if parents could discuss their coping strategies of peer pressure on a closed platform. However, the personal interaction between physicians, patients, and their parents still seems to promote compassionate psychological and medical attention and remains an important factor in the treatment cleft patients.

## Limitations

This study has some limitations. Firstly, it is retrospective in nature and therefore subject to confounding errors, such as varying memory due to widely differing follow-up periods between the participants. Secondly, substantially varying return rates were observed, which cannot satisfactorily be explained by the authors. International differing infrastructural reasons (e.g., possibly varying moving rates, varying convenience of postal service, varying rates of families with dual income, etc.) were assumed to cause this, but of course also differing satisfaction might influence return rates and bias findings, even though one would expect bigger international differences in overall satisfaction in this case. Also, we agree that further studies are needed to confirm our findings of intercultural differences and to work out the differences between several cleft centers and therapeutic strategies. Since NAM is a rather new treatment option, there are slight differences in the treatment plans from center to center, which may have evolved over time. Nevertheless, the investigated centers only differ very slightly in their strategies. Prospective multi-center studies would be highly desirable to strengthen the data in this field and subsequently apply the findings into even more sophisticated cleft care in all cultural areas. Nevertheless, in our opinion, this study was able to present reliable and valuable findings which can help to improve the current treatment protocols for both patients and their parents.

## Conclusion

Despite some significant intercultural differences between the parents of both cleft centers, all parents highly agreed that they were helping their children with NAM treatment, which proves that great efforts can result in great benefit not only for the children but also for the well-being of their parents. Nevertheless, the observed differences emphasize the need, as well as some possibilities of improvement in cleft treatment and parental support in the investigated countries, respectively, in the treatment of patients and parents with different cultural background.

## Data Availability

Due to the European Union General Data Protection Regulation, anonymized data is available on request from the corresponding authors.
